# Dietary triggers of gut inflammation following exclusive enteral nutrition in children with Crohn’s disease: a pilot study

**DOI:** 10.1186/s12876-021-02029-4

**Published:** 2021-12-03

**Authors:** Konstantinos Gkikas, Michael Logan, Ben Nichols, Umer Z. Ijaz, Clare M. Clark, Vaios Svolos, Lisa Gervais, Hazel Duncan, Vikki Garrick, Lee Curtis, Elaine Buchanan, Tracey Cardigan, Lawrence Armstrong, Caroline Delahunty, Diana M. Flynn, Andrew R. Barclay, Rachel Tayler, Simon Milling, Richard Hansen, Richard K. Russell, Konstantinos Gerasimidis

**Affiliations:** 1 Human Nutrition, School of Medicine, Dentistry and Nursing, College of Medical, Veterinary and Life Sciences, University of Glasgow, Glasgow, UK; 2 Civil Engineering, School of Engineering, University of Glasgow, Glasgow, UK; 3 Department of Paediatric Gastroenterology, Hepatology and Nutrition, Royal Hospital for Children, Glasgow, UK; 4 Department of Paediatrics, Crosshouse Hospital, Kilmarnock, UK; 5 Department of Paediatrics, Wishaw General Hospital, Wishaw, UK; 6 Institute for Infection, Immunity and Inflammation, University of Glasgow, Glasgow, UK; 7 Royal Hospital for Children and Young People, Edinburgh, UK

**Keywords:** Crohn’s disease, Food reintroduction, Dietary triggers, Faecal calprotectin, Gluten, Fibre, Meat, Short chain fatty acids

## Abstract

**Background:**

The anti-inflammatory effect of exclusive enteral nutrition on the gut of children with Crohn’s disease is rapidly lost after food reintroduction. This study assessed disease dietary triggers following successful treatment with exclusive enteral nutrition.

**Methods:**

Nutrient intake, dietary patterns and dietary biomarkers in faeces (gluten immunogenic peptides, undigestible starch, short chain fatty acids) were assessed in 14 children with Crohn’s disease during early food reintroduction, following exclusive enteral nutrition. Groups above (Group A) and below (Group B) the median levels of faecal calprotectin after food reintroduction were assigned for comparative analysis.

**Results:**

Intakes of fibre, gluten-containing cereals and red and processed meat were significantly higher in Group A than Group B; (median [Q1, Q3], g/day; Fibre: 12.1 [11.2, 19.9] vs. 9.9 [7.6, 12.1], *p* = 0.03; Red and processed meat: 151 [66.7, 190] vs. 63.3 [21.7, 67], *p* = 0.02; gluten-containing cereals: 289 [207, 402] vs. 203 [61, 232], *p* = 0.035). A diet consisting of cereals and meat products was predictive (92% accuracy) of higher faecal calprotectin levels after food reintroduction. In faeces, butyrate levels, expressed as absolute concentration and relative abundance, were higher in Group A than Group B by 28.4 µmol/g (*p* = 0.015) and 6.4% (*p* = 0.008), respectively. Levels of gluten immunogenic peptide and starch in faeces did not differ between the two groups.

**Conclusions:**

This pilot study identified potential dietary triggers of gut inflammation in children with Crohn’s disease after food reintroduction following treatment with exclusive enteral nutrition.

*Trial registration*: Clinical trials.gov registration number: NCT02341248; Clinical trials.gov URL: https://clinicaltrials.gov/ct2/show/NCT02341248 (retrospectively registered).

**Supplementary Information:**

The online version contains supplementary material available at 10.1186/s12876-021-02029-4.

## Introduction

The increasing incidence of Crohn’s disease (CD) in societies in economic transition, suggests that environmental factors, including a Western diet are major contributors to the disease pathogenesis [1]. In nutritional epidemiology, adherence to the principles of the Mediterranean diet has protected against development of CD [2, 3] whereas food additives have been implicated in the development of intestinal inflammation in animal models [4].

Exclusive enteral nutrition (EEN) is the only established dietary treatment for active CD in children [5, 6]. In addition to symptom improvement, EEN reduces faecal calprotectin (FC) levels by a mean of 50% by the end of treatment [7]. However, we have recently observed a rapid increase in FC within the first 17 days of food reintroduction, following treatment with EEN; an effect which preceded any noticeable changes in clinical disease activity [8]. This intriguing observation suggests that reintroduction of certain dietary components, after return to habitual diet, provokes recurrence of intestinal inflammation.

In the current study, we performed detailed assessment of the diet of children with CD during this early phase of food reintroduction, following successful EEN, and explored relationships with FC. We concentrated our efforts on this critical food reintroduction period, which provided us with a unique timeframe to explore dietary disease triggers in a population with homogeneous characteristics of disease activity, type, and duration of preceding EEN treatment at study enrolment.

## Methods

### Patients

Children (aged 3–17 years) with a new diagnosis of CD or with disease in relapse, who initiated an 8-week course of EEN were recruited from the Royal Hospital for Children, Glasgow and neighbouring hospitals, as described previously [9]. A faecal sample per patient was collected during early food reintroduction, between 15 and 30 days after EEN completion or at the earliest most convenient time for the patients.

Disease activity was assessed with the weighted Paediatric Crohn’s Disease Activity Index (wPCDAI) [10]. In the current study, we included a subset of patients recruited in the study by Logan et al. [9], who achieved both clinical remission at EEN completion (wPCDAI < 12.5) and experienced a clinically significant drop in FC, defined by a decrease of ≥ 50% or ≥ 500 mg/kg from levels at EEN initiation (Additional file 1: Fig. S1). Patients who did not enter clinical remission or did not provide a faecal sample or dietary records were excluded. Information about disease phenotype (Paris classification) [11] and concomitant treatment was collected from medical notes.

### Dietary assessment

Participants recorded their diet for three days prior to faecal sample collection, including intake of enteral nutrition as maintenance treatment (MEN), with 3-day food diaries, estimating weight of consumed foods using household measures. MEN was prescribed to provide 20–25% of daily energy requirements. No specific food reintroduction guidance was provided to patients after EEN cessation. Dietary records were analysed using the WinDiets software (WinDiets version 10, Robert Gordon University, Aberdeen, UK) for calculation of energy and nutrient intake.

Dietary patterns were assessed by classification of individual foods in groups based on the grouping applied in the UK National Dietary and Nutrition Survey (NDNS) [12]. Individual foods were assigned to the subsidiary NDNS food groups (level 3, e.g., rice) and the subsidiary groups were combined to form the main NDNS groups (level 2, e.g., pasta, rice, and other cereals), which then formed the larger level food groups (e.g., cereal and cereal products) (level 1) (Additional file 2: Fig. S2). Cereal products were divided to gluten and non-gluten containing cereal products. Meat subtypes were also grouped under the categories: ‘red meat’ and ‘processed meat’ separately, and also in a combined ‘red and processed meat’ category based on the World Cancer Research Fund definitions [13], since the consumption of both types of meat has been implicated in the pathogenesis of Inflammatory bowel diseases (IBD) [14, 15]. Nutrient intake was expressed in absolute mass (grams). Energy intake was further expressed as percentage of estimated average requirements (EAR), macronutrients as percentages of total energy intake, and protein and micronutrients as percentages of reference nutrient intakes (RNI) [16]. Dietary fibre was defined as non-starch polysaccharides, in accordance with the Englyst method.

### Faecal sample collection and faecal calprotectin

The entire bowel movement was collected within 4 h of defecation and was transferred to the laboratory on ice under anaerobic conditions (Oxoid AnaeroGen Sachet; ThermoFisher Scientific)[9]. Samples were homogenised and aliquots were stored in – 80 °C. Faecal calprotectin was measured using the CALP0170 kit (CalproLab, Lysaker, Norway) [17].

### Faecal biomarkers of food intake

Short and branched chain fatty acids (SCFA, BCFA) were measured using gas chromatography (Agilent 7890A) [18], and were used as proxy biomarkers of fermentable dietary fibre and protein intake, respectively. Starch (Megazyme, Ireland) and gluten immunogenic peptides (GIP, Biomedal, S.L., Spain) were measured in faeces, as biomarkers of non-fermented resistant starch and gluten intake, respectively [19], according to the manufacturers’ instructions. For the measurement of faecal starch, samples were initially lyophilised and homogenised thoroughly using a pestle and mortar.

### Statistical considerations

Statistical analysis was performed using Minitab, Version 18 (Minitab Ltd, Coventry, UK) and R (version 3.5.3). Patients were divided into two equally numbered groups based on their FC levels during food reintroduction. Patients with an FC above the median concentration after food reintroduction were assigned to Group A (Above) and patients with an FC below the median concentration to Group B (Below). Continuous data were expressed as medians with interquartile ranges (Q1, Q3) and categorical data as counts with frequencies (n [%]), unless otherwise stated. Comparisons of continuous variables between different groups were performed on Box-Cox transformed data using general linear models and Fisher’s least significance difference post-hoc test. Comparisons of dietary intake and biomarker levels between the two groups were adjusted for FC levels at EEN completion (p-adj). Relationships between dietary components and FC levels were explored with Pearson and Spearman rank correlations.

For dietary pattern analysis, subset regression analysis was performed by testing all possible food group combinations. The best predictive model was selected according to recommendations given in Kassambara, 2018 [20], and was used to predict the FC group of each patient based on dietary patterns consisting of multiple food groups. In-house R scripts incorporating the *leaps* [21] and *caret* packages [22], were used. Specifically, we used the relative contribution of each individual food group intake to the cumulative food group intake. Food groups consumed by fewer than two patients were excluded from the analysis. After the various models were created, random forest analysis, using the *randomForest* package [23] was employed to select the best model based on the lowest assignment error (out-of-bag error). The relative importance of each food group intake to the predictive ability of each model was assessed by calculating the mean reduction in Gini impurity index on inclusion.

## Results

### Anthropometric and clinical disease characteristics

Fourteen out of 23 (61%) paediatric patients, who had maintained symptom-free clinical remission at the time of the sample collection, provided a faecal sample and completed dietary records within (median [Q1, Q3]): 21 [15, 51] days of food reintroduction. None had commenced or were receiving another induction therapy. The median [Q1, Q3] FC for all 14 patients after food reintroduction was 900 mg/kg [341, 1243]. In Group A, median [Q1, Q3] FC levels were 1,181 [1024, 1781] mg/kg and in Group B 411 [130, 651] mg/kg (Additional file 3: Fig. S3). No significant differences were observed in demographics, anthropometry, or disease characteristics between the two groups (Additional file 4: Table S1). There were no significant differences in immunomodulator use, energy intake from MEN, and the time elapsed between the end of EEN and sample collection after food reintroduction. Likewise, no significant correlation was observed between FC levels and the time elapsed between the end of EEN and sample collection after food reintroduction (*p* = 0.899).

### Nutrient intake

Group A reported a significantly higher intake of fibre compared to Group B, expressed both as an absolute amount (median [Q1, Q3], grams; Group A: 12.1 [11.2, 19.9] vs. Group B: 9.9 [7.6, 12.1], *p* = 0.030, *p*-adj = 0.068) and as percentage of RNI (median [Q1, Q3], %RNI; Group A: 55 [46, 99.5] versus Group B: 44.8 [30.3, 48.5], *p* = 0.023, *p*-adj = 0.071) (Table 1). Intakes of protein and phosphorus, expressed as %RNI, were also significantly higher in Group A than Group B (median [Q1, Q3], %RNI; Protein; Group A: 262 [195, 291] versus Group B: 211 [88.4, 215], *p* = 0.026, *p*-adj = 0.069; Phosphorus; Group A: 241 [171, 290] versus Group B: 177 [130, 191], *p* = 0.040, *p*-adj = 0.174) (Table 1).

**Table 1:** Comparison of nutrient intake between patients in the two groups

	Group A (n = 7)	Group B (n = 7)	*p* value	*p* value adj
Total energy (kcal)	2057 (1916, 2305)	1906 (1473, 2252)	0.365	0.217
Total energy (% EAR)	97.2 (89.2, 118)	85.7 (69.3, 105)	0.08	0.177
Fat (g)	71.6 (65.7, 82.5)	75.3 (63, 83.6)	0.764	0.719
Fat (% kcal)	32.3 (28.4, 35.5)	33.8 (30.6, 35.6)	0.418	0.317
SFA (g)	25.0 (24.3, 33.1)	29.0 (24.8, 37.4)	0.602	0.533
SFA (% kcal)	11.7 (10.5, 13.9)	13.1 (10.9, 15.2)	0.381	0.269
MUFA (g)	27.1 (20.9, 27.7)	21.8 (17.5, 24.5)	0.237	0.315
MUFA (% kcal)	10.9 (9.8, 12.0)	10.5 (9.7, 11.5)	0.463	0.78
PUFA (g)	11.5 (9.8, 12.9)	9.4 (6.9, 10.8)	0.082	0.236
PUFA (% kcal)	4.8 (3.8, 6.5)	4.3 (4.1, 5.1)	0.336	0.887
Carbohydrates (g)	267 (239, 303)	246 (225, 284)	0.415	0.19
Carbohydrates (% kcal)	52.5 (48.5, 56.0)	51.6 (49, 56.9)	0.831	0.807
Sugars (g)	97.4 (84.1, 105)	93.3 (89.1, 118)	0.636	0.393
Sugars (% kcal)	18.9 (16.2, 22)	21.4 (20.9, 24.2)	0.175	0.393
Dietary fibre (g)	12.1 (11.2, 19.9)	9.9 (7.6, 12.1)	0.03	0.068
Dietary fibre (% RNI)	55 (46, 99.5)	44.8 (30.3, 48.5)	0.023	0.071
Starch (g)	146 (105, 154)	126 (120, 144)	0.366	0.216
Protein (g)	82.2 (73.7, 90.2)	65.4 (37.2, 89.5)	0.179	0.159
Protein (% kcal)	16.8 (13.8, 17.3)	14.9 (10.7, 15.9)	0.135	0.196
Protein (% RNI)	262 (195, 291)	211 (88.4, 215)	0.026	0.069
Vitamin A (µg)	485 (451, 762)	695 (485, 1000)	0.709	0.464
Vitamin A (% RNI)	80.8 (75.2, 153)	139 (80.8, 167)	0.948	0.617
Vitamin D (µg)	1.2 (0.9, 1.6)	0.9 (0.6, 1.2)	0.536	0.844
Vitamin C (mg)	115 (62.1, 127)	102 (77.6, 117)	0.909	0.871
Vitamin C (% RNI)	362 (207, 401)	291 (194, 389)	0.821	0.743
Thiamine (mg)	1.8 (1.2, 2.2)	1.4 (0.8, 1.9)	0.297	0.483
Thiamine (% RNI)	251 (171, 310)	159 (101, 212)	0.068	0.214
Vitamin B2 (mg)	1.3 (1.1, 1.7)	1.4 (1, 1.8)	0.608	0.352
Vitamin B2 (% RNI)	141 (112, 174)	129 (103, 153)	0.343	0.404
Vitamin B6 (mg)	2.3 (1.6, 2.8)	2.2 (1.7, 3.2)	0.938	0.548
Vitamin B6 (% RNI)	234 (141, 276)	184 (145, 267)	0.63	0.772
Niacin (mg)	34.3 (26.8, 42.5)	32.3 (25.8, 37.3)	0.338	0.323
Niacin (% RNI)	283 (224, 354)	231 (172, 249)	0.104	0.143
Folic acid (µg)	215 (163, 306)	211 (172, 294)	0.784	0.71
Folic acid (% RNI)	139 (81.5, 204)	106 (86, 147)	0.374	0.859
Vitamin B12 (µg)	3.4 (2.9, 4.9)	3.6 (2.8, 4.9)	0.89	0.472
Vitamin B12 (% RNI)	337 (195, 412)	324 (187, 414)	0.828	0.567
Pantothenic acid (mg)	4.8 (4.2, 5.7)	4.2 (3.2, 6.6)	0.454	0.708
Biotin (µg)	24 (22.1, 27)	22.1 (16.2, 39)	0.893	0.357
Vitamin E (mg)	6.4 (3.6, 7.6)	6.1 (4.5, 6.5)	0.816	0.717
Sodium (mg)	2084 (1977, 2834)	2073 (1399, 2682)	0.528	0.365
Sodium (% RNI)	130 (124, 177)	130 (87.4, 168)	0.528	0.365
Chloride (mg)	3307 (2368, 4315)	3647 (2180, 3756)	0.484	0.49
Chloride (% RNI)	167 (94.7, 238)	146 (121, 150)	0.244	0.362
Potassium (mg)	2293 (1930, 3068)	2326 (1807, 2617)	0.468	0.651
Potassium (% RNI)	115 (99, 223)	84.4 (72.4, 96)	0.127	0.4
Calcium (mg)	1068 (718, 1323)	1103 (645, 1470)	0.942	0.951
Calcium (% RNI)	128 (93.3, 157.2)	102 (79.6, 115)	0.142	0.571
Phosphorus (mg)	1241 (1005, 1323)	1103 (814, 1470)	0.527	0.821
Phosphorus (% RNI)	241 (171, 290)	177 (130, 191)	0.04	0.174
Magnesium (mg)	262 (187, 285)	207 (179, 270)	0.53	0.981
Magnesium (% RNI)	101 (66.8, 142)	82.3 (68.8, 90.1)	0.218	0.692
Iron (mg)	10.9 (7.9, 14.1)	8.9 (8.2, 12.7)	0.414	0.979
Iron (% RNI)	114 (69.6, 148)	76.4 (73.5, 112)	0.257	0.886
Zinc (mg)	11.4 (6.70, 13.8)	7.8 (6.35, 10.2)	0.393	0.745
Zinc (% RNI)	153 (95.7, 164)	106 (86.7, 117)	0.179	0.474
Copper (mg)	0.99 (0.95, 1.55)	1.07 (0.76, 1.26)	0.443	0.857
Copper (% RNI)	136 (95, 221)	111 (95.4, 134)	0.268	0.964
Iodine (µg)	91.7 (80, 117)	117 (90.3, 162)	0.468	0.322
Iodine (% RNI)	77.4 (70.5, 106)	102 (64.5, 124)	0.811	0.46
Selenium (µg)	42 (31, 49.3)	30 (24, 54.7)	0.382	0.762
Selenium (% RNI)	110 (67.4, 153)	73.3 (44.4, 121)	0.159	0.531
Cholesterol (mg)	208 (160, 286)	203 (73, 268)	0.464	0.506
Carotene (ug)	1561 (325, 3497)	950 (179, 1939)	0.370	0.415

Group A: patients with faecal calprotectin levels above the median levels at food reintroduction, Group B: patients with faecal calprotectin values below the median levels at food reintroduction. *p* value adj: p value adjusted for faecal calprotectin levels at the end of EEN. Values are presented as medians (Q1, Q3). EAR: Estimated average requirement, MUFA: Monounsaturated fatty acids, PUFA: Polyunsaturated fatty acids, RNI: Reference nutrient intake, SFA: Saturated fatty acids

### Food group intake

**Fig. 1:**
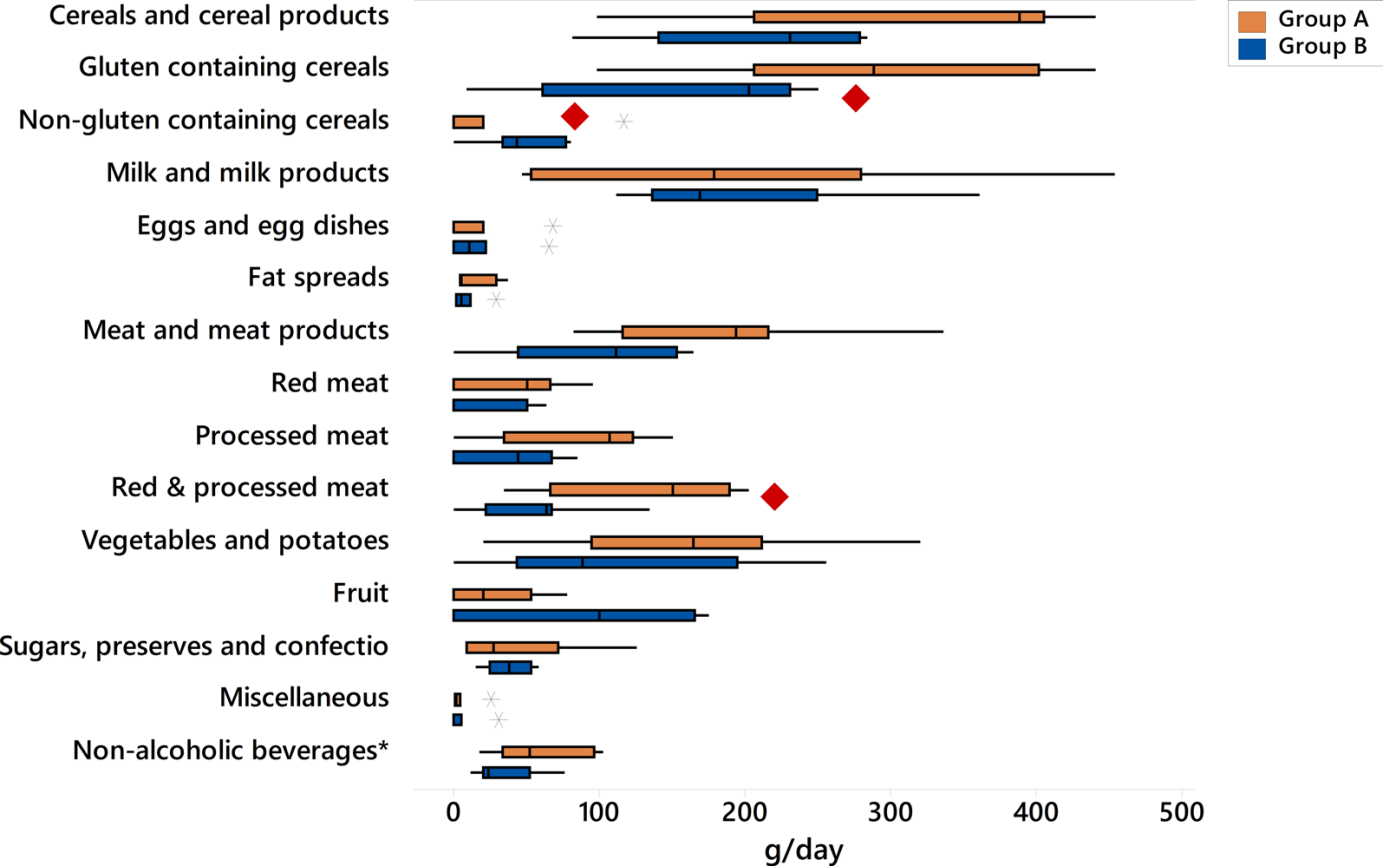
Comparison of food group intake between the two groups in children with Crohn's Disease during food reintroduction. Group A: patients with faecal calprotectin levels above the median levels at food reintroduction, Group B: patients with faecal calprotectin values below the median levels at food reintroduction * Intake of non-alcoholic beverages has been divided by 10 for both groups for better visualisation. Red diamond indicates significant differences between Group A and B (*p* = 0.03)

Although the intake of red meat and processed meat, when assessed separately, did not differ between the two groups (*p* = 0.225, *p* = 0.125 respectively), their cumulative intake in Group A was significantly higher compared to Group B by a median difference of 87.7 g/day (median [Q1, Q3], grams; Group A: 151 [66.7, 190] vs. Group B: 63.3 [21.7, 67], *p* = 0.030, *p*-adj = 0.065) (Fig. 1). Consumption of cereals and cereal products was higher in Group A, although the difference did not reach statistical significance (*p* = 0.08, *p*-adj = 0.067). Nonetheless, the intake of gluten containing cereal products was higher in Group A than Group B, and in reverse, the consumption of non-gluten containing cereal products was higher in Group B (median [Q1, Q3], grams; gluten containing cereal products, Group A: 289 [207, 402] vs. Group B: 203 [61, 231], *p* = 0.035, *p*-adj = 0.042; non-gluten containing cereal products: Group A: 0 [0, 20] vs. Group B: 43 [33, 77], *p* = 0.031, *p*-adj = 0.083). The food groups ‘alcoholic beverages’, ‘commercial toddlers’ foods and drinks’ and ‘nuts and seeds’ were not consumed by any of the patients and were therefore excluded from analysis.

### Relationships between nutrients, food groups, dietary patterns, and levels of FC

**Fig. 2:**
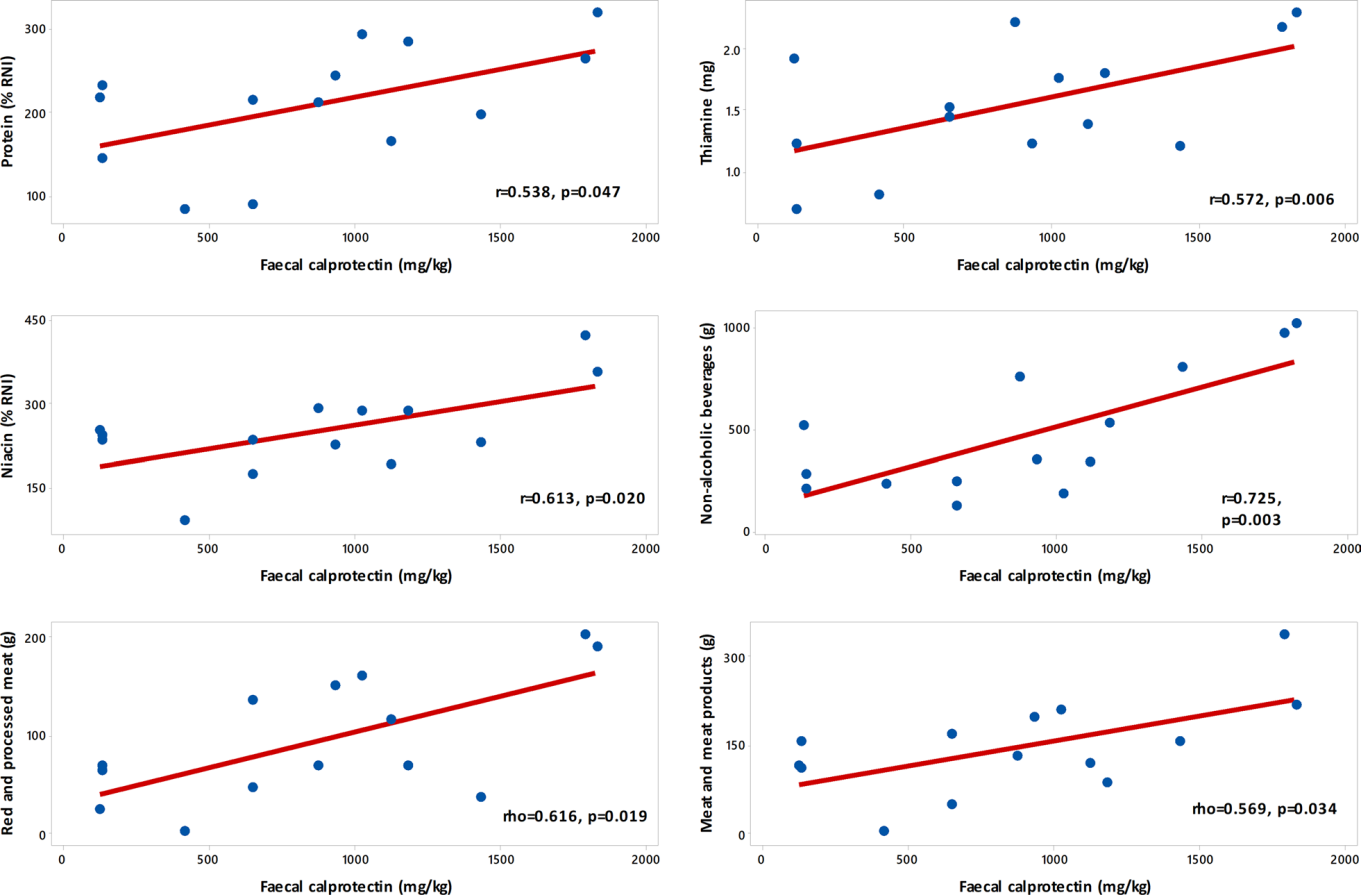
Correlations between nutrient and food intake with faecal calprotectin levels in children with Crohn's Disease after food reintroduction

To explore linear relationships, as a proxy of dose–response associations between dietary intake parameters and FC levels, we performed correlation analysis (Fig. 2, Additional file 4: Tables S2 and S3). Protein intake (% RNI) (r = 0.54, *p* = 0.047), thiamine (mg) (Pearson r = 0.57, *p* = 0.033) and niacin (%RNI) (r = 0.61, p = 0.02) had a moderate-strong positive correlation with FC (Fig. 2).

Moderate-strong significant positive correlations with FC levels were also observed between the intakes of red and processed meat (Spearman’s rho = 0.62, *p* = 0.019), meat and meat products (Spearman’s rho = 0.57, *p* = 0.034) and the intake of non-alcoholic beverages which included juices, and fizzy drinks (Pearson r = 0.73, *p* = 0.003) (Fig. 2).

**Fig. 3:**
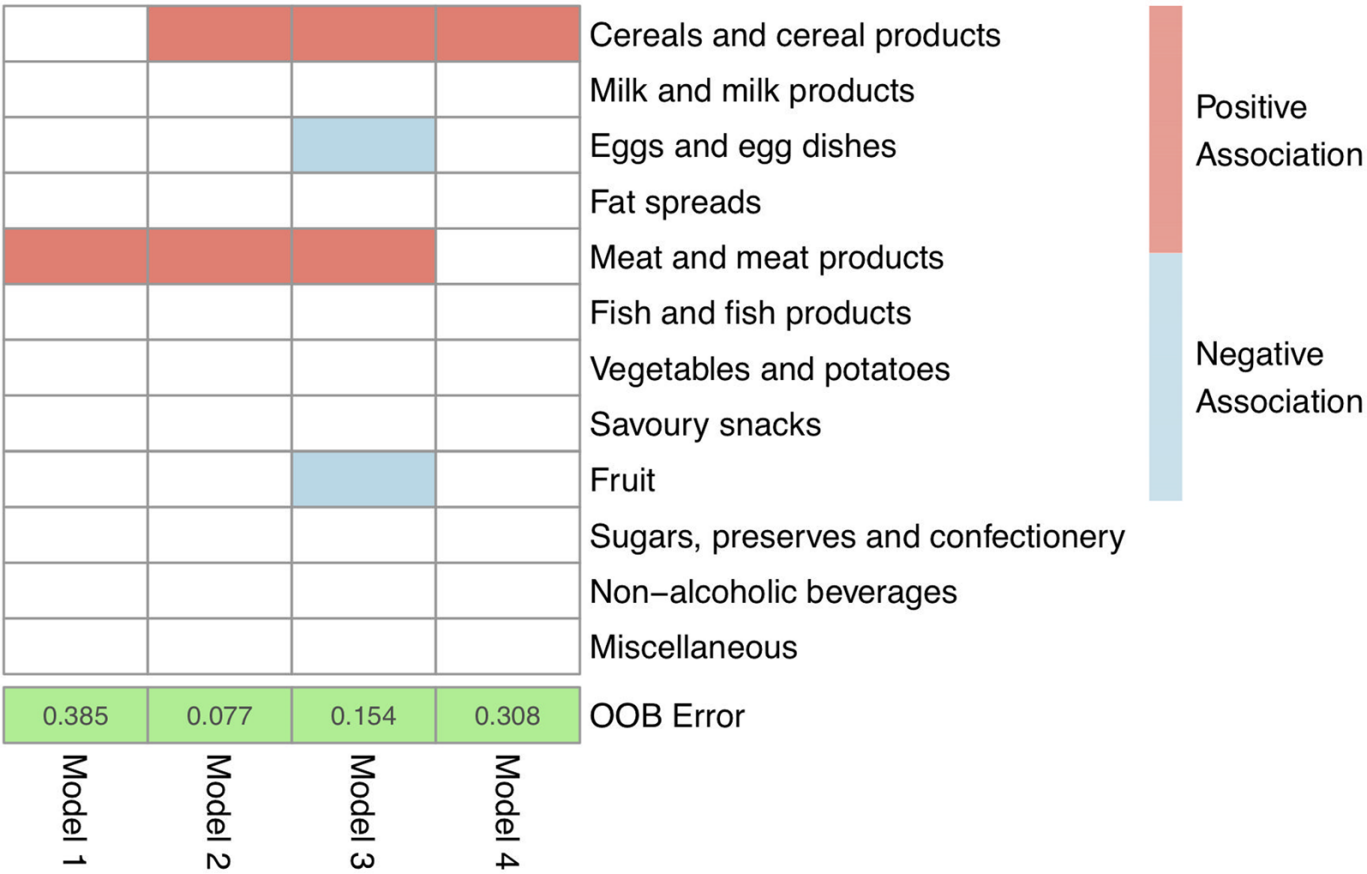
Results from subset regression analysis showing food-group models predicting classification of patients in the two FC groups. Food groups coloured with red indicate positive association (i.e., harmful) and with blue negative (i.e., beneficial) association with assignment of patients in the group with higher FC levels during food reintroduction (Group A) (higher FC levels during food reintroduction)**.** OOB error: Out of bag error rate, showing percentage of misclassification of patients to their respective FC groups (Group A/B) for each different food-group model identified from random forest analysis. Fish and fish-related products were removed from analysis, as they were consumed by one patient

The contribution of dietary patterns in the prediction of FC levels was assessed using food-group models (Fig. 3). Cereals and meat products showed a positive association with the assignment of patients in Group A, whilst for eggs, fruits and fruit products, the association was negative. Random forest analysis showed that the model with the lowest ‘out-of-bag’ error included cereal, meat and their products, and could predict the correct classification of patients to their respective FC group with a high accuracy of over 92% (error rate: 7.7%, Model 2, Fig. 3). The contribution of meat and meat products to the predictive ability of Model 2 was 62%, and that of cereals 38%, suggesting that a dietary pattern high in cereals and meat products was associated with higher levels of FC after food reintroduction. The model with the second lowest error rate included in addition fruits and eggs, which showed a negative (i.e., a protective) association with faecal calprotectin levels (error rate: 15.4%, Model 3, Fig. 3).

### Diet-related biomarkers in faeces

Butyrate levels, expressed either as absolute concentration (µmol/g) or as relative proportion (%), were significantly higher in patients with higher FC levels (Group A), compared to patients with lower FC levels (Group B) by a median difference of 28.4 µmol/g and 6.4% respectively (median [Q1, Q3], µmol/g: Group A: 62.5 [41.8, 114] vs. Group B: 34.1 [29.2, 49.6], *p* = 0.016, *p*-adj = 0.015; %; Group A: 15.1 [11.2, 17.8] vs. Group B: 8.7 [8.6, 9.12], *p* = 0.008, *p*-adj = 0.016), Table 2. The concentration of acetate, propionate and of BCFA did not differ between the two groups. Faecal GIP levels did not significantly differ between the two groups (median [Q1, Q3], ng/µg; Group A: 1,250 [1,250, 1,250] vs. Group B: 1,250 [78, 1,250], *p* = 0.121, *p*-adj = 0.211), although most patients in Group A had GIP measurements above the upper detection limit of the assay, suggesting high gluten intake. There was no difference in faecal starch output between the two groups (median [Q1, Q3], g/100 g; Group A: 0.2 [0.1, 0.8] vs. Group B: 1.10 [0.10, 1.40], *p* = 0.141, *p*-adj = 0.573).

**Table 2:** Comparison of concentration of SCFA in faeces between patients in the two groups

SCFA	Group A (n = 6)	Group B (n = 7)	*p* value	*p* value adj
Faecal water content (%)	74 (70, 80.5)	73.2 (65.7, 80)	0.601	0.340
Acetate (µmol/g)	255 (238, 341)	304 (223, 385)	0.716	0.959
Propionate (µmol/g)	80.5 (70.5, 143)	72.1 (46.3, 109)	0.367	0.126
Isobutyrate (µmol/g)	10.9 (7.6, 16.7)	8.70 (6.1, 9.9)	0.121	0.174
Butyrate (µmol/g)	62.5 (41.8, 114)	34.1 (29.2, 49.6)	0.016	0.015
Isovalerate (µmol/g)	11.4 (7, 17.4)	8.6 (4.8, 11.7)	0.180	0.297
Total SCFA (µmol/g)	401 (386, 663)	395 (344, 537)	0.533	0.255
Acetate (%)	60.2 (56.7, 65.2)	71.6 (60.2, 75.4)	0.037	0.027
Propionate (%)	20.6 (18, 21.4)	18.7 (12.4, 20.9)	0.450	0.208
Isobutyrate (%)	2.2 (2.02, 3.4)	1.9 (1.4, 2.5)	0.163	0.355
Butyrate (%)	15.1 (11.2, 17.8)	8.6 (8.6, 9.1)	0.008	0.016
Isovalerate (%)	2.1 (1.9, 3.8)	1.8 (1.2, 3.1)	0.295	0.598

Group A: patients with faecal calprotectin levels above the median levels at food reintroduction, Group B: patients with faecal calprotectin values below the median levels at food reintroduction. Values are presented as medians (Q1, Q3). *p* value adj: *p* value adjusted for faecal calprotectin levels at the end of EEN. %: proportional ratio of each SCFA to total SCFA; SCFA: Short chain fatty acids

## Discussion

This study identified differences in the intake of certain nutrients, dietary patterns, and diet-related biomarkers in faeces between children with CD who demonstrated different levels of intestinal inflammation after food reintroduction, following successful treatment with EEN.

A higher intake of fibre and of butyrate, its proxy biomarker in faeces, was observed in patients with raised levels of FC. This observation is in contrast to the previously ascribed protective role of fibre in the development of CD [3, 24, 25]. Likewise, the role of fibre in the management of IBD remains unclear. While observational research data point to a protective role of fibre in preventing a disease flare in CD [26], other data showed that a higher dietary fibre intake was positively associated with risk for clinical relapse [27]. Overall, the findings of the current study align with the lack of evidence supporting the effectiveness of fibre in the management of the disease [28–31]. A compositional analysis of 61 EEN formulas used for the induction of remission in CD also showed that < 20% of those formulas contained fibre [32]. Collectively, these results demonstrate that lack of fibre does not have a deleterious effect on disease activity, and indeed may have a seemingly unexpected unfavourable effect.

Patients in the current study with higher FC levels reported a higher protein and phosphorus intake, along with a higher intake of red and processed meat. Although we did not assess separately the intake of protein from animal or plant sources, the presence of a moderate-to-strong correlation between the intake of protein and meat products (spearman rho = 0.66, *p* = 0.01), but not with cereal products, nor vegetables indicates that protein from animal sources is likely to explain the negative relationship with FC we observed in the current study. High intake of animal protein has been associated with development of IBD [33, 34], and in patients with ulcerative colitis (UC), high intake of protein, total, as well as red and processed meat, was positively associated with risk of relapse [15]. In a recent retrospective study, patients with IBD in clinical remission had a lower total and animal protein intake compared to patients who experienced a relapse after a 2-year follow-up [35]. In contrast, a recent RCT showed that clinical relapse rates and FC levels did not differ between patients with CD who consumed at least two portions of red and processed meat per week, and others who consumed less than one serving per month [36]. The positive associations between FC and phosphorus intake may indicate an increased intake of grains and meat products which are rich in phosphorus. Although the effect of phosphorus has not been extensively explored in human IBD, dietary phosphate has been shown to be pro-inflammatory in animals [37].

Using machine learning on food group-based analysis, we showed that a dietary pattern consisting of cereal and meat-based products could successfully predict the assignment of 92% of patients into their respective FC group, with meat and cereal products showing a positive association with FC. On the contrary, the intake of eggs and fruit was associated with lower FC levels in another model with higher misclassification error though. In a recent cross-sectional study of patients with CD, principal component analysis also identified that a dietary pattern rich in rice, pasta and red meat, among other foods, was associated with increased symptom frequency, but did not differentiate patients with active disease from those in remission [38]. In another study which assessed dietary patterns, a “Western-type” eating pattern consisting of mainly grains, red and processed meat and high-sugar foods was positively associated with a higher risk for clinical relapse [35]. Of note, most novel dietary therapies for management of disease activity and intestinal inflammation in patients with CD often exclude or limit the intake of grains/gluten and red and processed meat [39]. In the current study, patients with higher FC levels also reported a higher consumption of gluten containing cereal products, which points to gluten-containing foods as potential driver of intestinal inflammation.

In addition to conventional dietary assessment, we measured diet-originating bacterial metabolites and dietary components in faeces as complementary biomarkers of consumption of certain foods previously implicated in CD pathogenesis [40]. The lack of significant differences in the levels of faecal starch between the two CD groups did not parallel the signals we observed with fibre intake. However, fibre encompasses an umbrella term of structurally diverse carbohydrates with potentially different roles in CD [41]. Although the intake of gluten containing cereals was higher in Group A, faecal GIP levels did not differ between the two groups. However, the method used to quantify faecal GIP is sensitive at detecting transgressions to gluten-free diet compliance rather than quantitatively estimating variable intakes of gluten, which might be more important in a dose–response relationship with initiation of intestinal inflammation [19].

In the current study, double levels of faecal butyrate levels were observed in patients with higher FC levels after food reintroduction. This signal was associated with higher dietary fibre intake from which butyrate originates as an end-product of bacterial fermentation in the same group (Spearman’s correlation between fibre and butyrate levels: ρ = 0.55, *p* = 0.051) [42]. Higher levels of butyrate in the caecum have been shown to aggravate animal colitis [41] and a significant reduction in butyrate levels in faecal samples of children responding to EEN paralleled with a decrease in FC [18, 43]. These data suggest that high amounts of butyrate are not of crucial importance for maintenance of intestinal health in patients with CD and its role in the disease course requires further exploration.

Although patients in Group A reported higher protein intakes compared to those in Group B, there were no significant differences in BCFA levels between the two groups. This could potentially result from adequate intake of dietary fibre in those patients, which exceeds the threshold below which excessive protein fermentation occurs in the colon [17].

The small sample size is the main limitation of this pilot study. This was due to the modest number of patients who met our stringent inclusion criteria and few patients who did not return dietary records paired with faecal samples. However, selection of a homogenous population with all patients enrolled at the end of an EEN course and while they were still in clinical remission, without receiving other concomitant induction treatment, minimised variance in our measured outcomes and increased statistical power. Beyond the dietary analysis presented here, the observed dietary signals of the current study might also represent biomarkers of unidentified covariates of other food ingredients which might be important in gut inflammation in CD. An indicative example could be baker’s yeast, which is present in bread and bakery products, constitutes a significant proportion of the cereal and cereal-based products group, and has been previously implicated in aggravating disease activity in CD [44]. It is also possible that nutrient interactions are more important than single nutrients alone in the underlying pathogenesis of CD [45]. Measurement of SCFA in faeces is the abstract of net production and absorption. However, as direct measurement of SCFA in the caecum would be almost impossible, faecal SCFA are considered suitable proxies of intestinal SCFA production; and by extension of fibre intake as has been demonstrated here by the positive associations between the amount of fibre consumed and levels of faecal SCFA [46, 47].

## Conclusions

The current pilot study for first time in the literature identified that a diet higher in dietary fibre, protein, and red processed meat was associated with higher levels of intestinal inflammation after early food reintroduction in children with CD, post-EEN. These data need confirmation in larger, prospective studies to gain more insight into the dietary triggers of gut inflammation in CD, including the role subtypes of dietary fibre, and any mediating role the microbiome may have. Such research would help identify dietary components causing inflammation in CD and enable development of food reintroduction and personalised dietary regimes, leading potentially to improvements in CD management and prolonged clinical remission. A clinical trial like that is currently undergoing [48].

## Supplementary Information

**Additional file 1: Fig. S1** (.png) Faecal calprotectin values before the start of EEN treatment and at 4 weeks and 8 weeks of EEN treatment in all 14 patients. Different colours indicate different patients.

**Additional file 2: Fig. S2**. (.pdf) Hierarchical dendrogram showing the classification of individual food groups to larger level food groups, based on the National Diet and Nutrition Survey

**Additional file 3: Fig. S3**. (.tif) Stratification of patients based on the median faecal calprotectin (900 mg/kg) of the entire group at food reintroduction. Group A (n=7): above median faecal calprotectin, Group B (n=7): below median faecal calprotectin.

**Additional file 4.** Supplementary Tables 1, 2, 3.

## Data Availability

The data underlying this article will be shared on reasonable request to the corresponding author.

## Abbreviations

BCFA: Branched chain fatty acids
FC: Faecal calprotectin
GIP: Gluten immunogenic peptides
NDNS: National Diet and Nutrition Survey
SCFA: Short chain fatty acids

## Declarations

### Guidelines statement

All methods were carried out in accordance with relevant guidelines and regulations.
