# Supplementary figures and images for: Dietary triggers of gut inflammation following exclusive enteral nutrition in children with Crohn’s disease: a pilot study
Source: BMC Gastroenterol. 2021 Dec 3;21:454. doi: 10.1186/s12876-021-02029-4 (PMC8642954; doi:10.1186/s12876-021-02029-4)

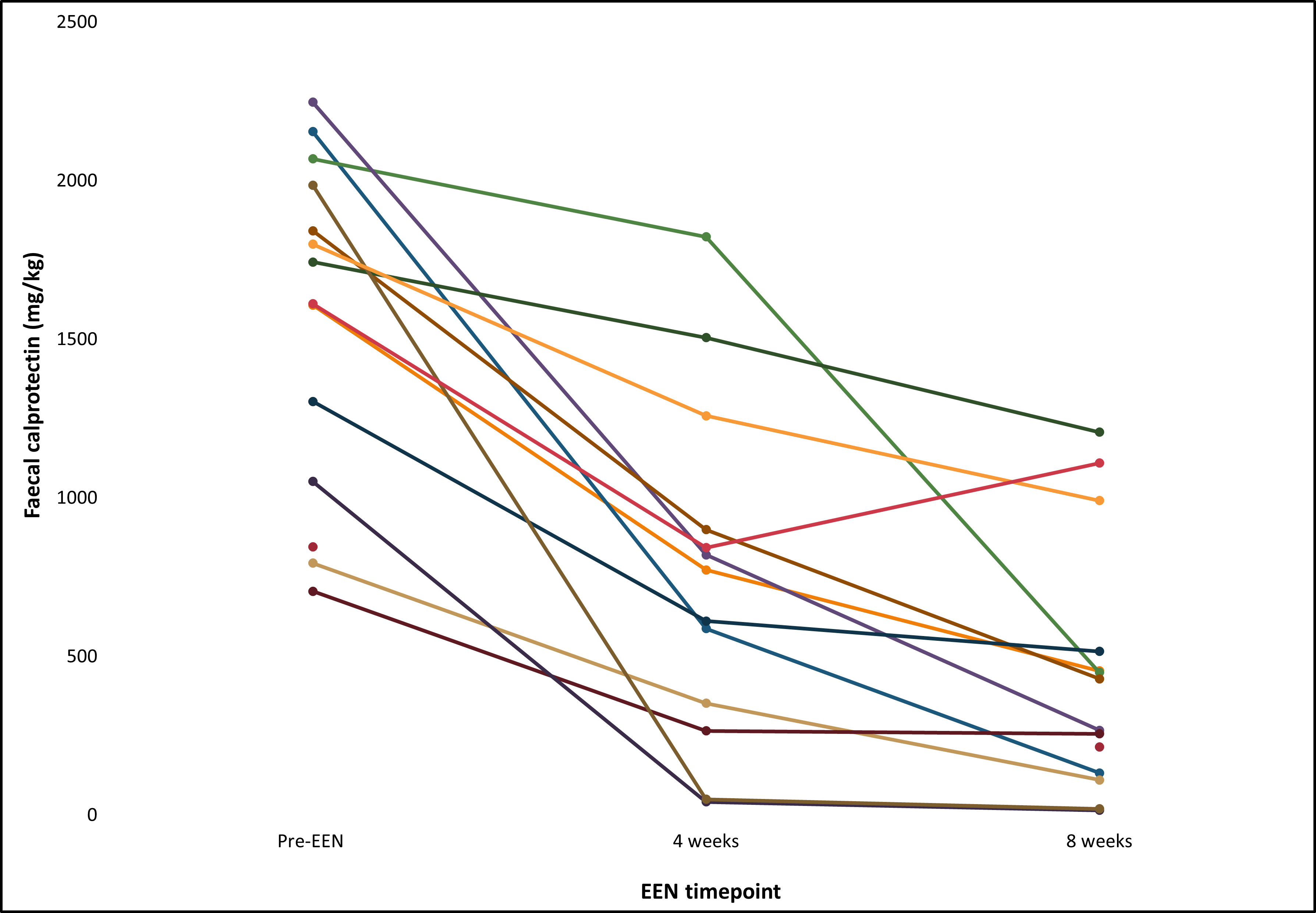

Supplement: Supplementary file 1 — Additional file 1: Fig. S1 (.png) Faecal calprotectin values before the start of EEN treatment and at 4 weeks and 8 weeks of EEN treatment in all 14 patients. Different colours indicate different patients. [file 12876_2021_2029_MOESM1_ESM.png]

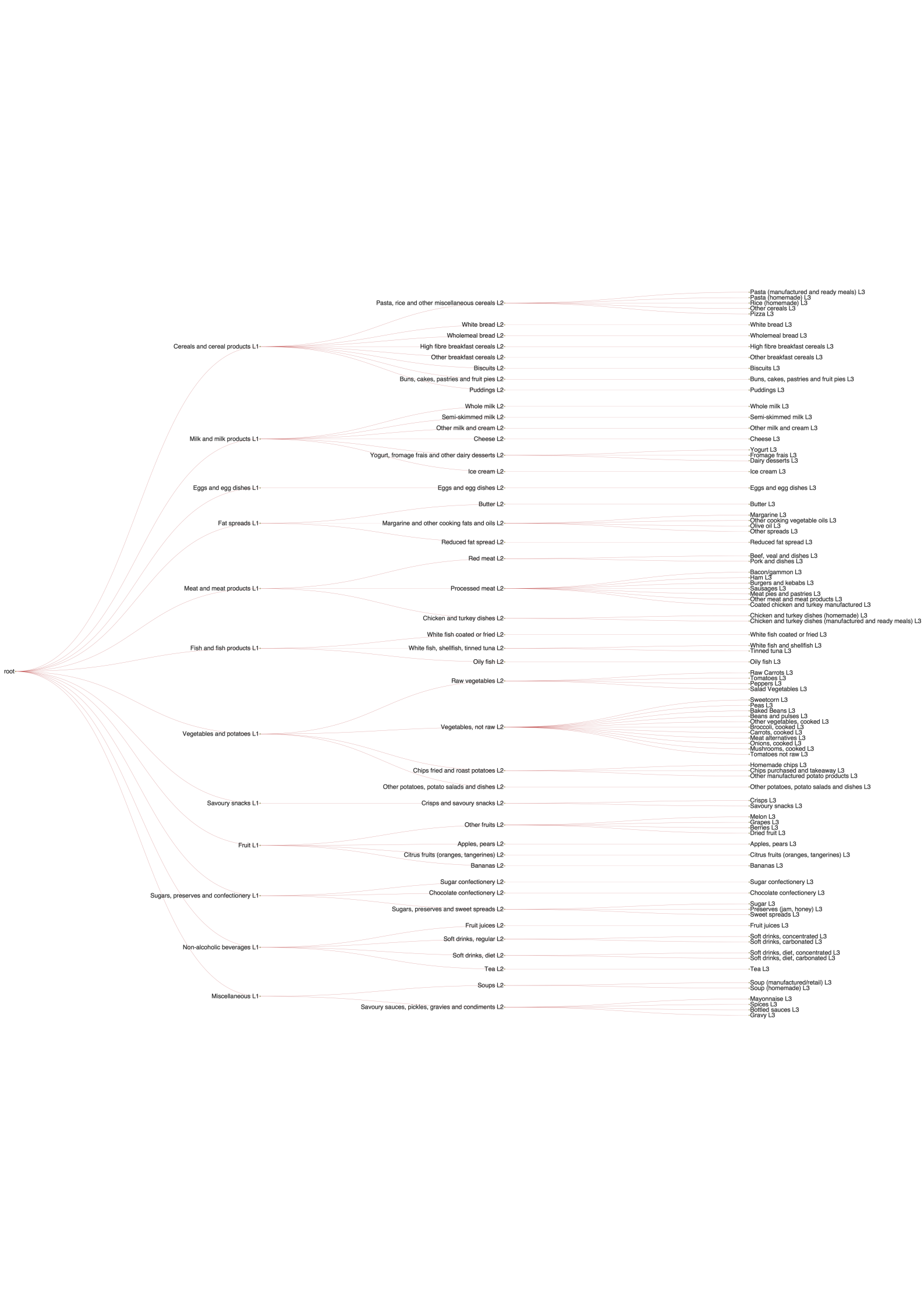

Supplement: Supplementary file 2 — Additional file 2: Fig. S2. (.pdf) Hierarchical dendrogram showing the classification of individual food groups to larger level food groups, based on the National Diet and Nutrition Survey [file 12876_2021_2029_MOESM2_ESM.pdf]

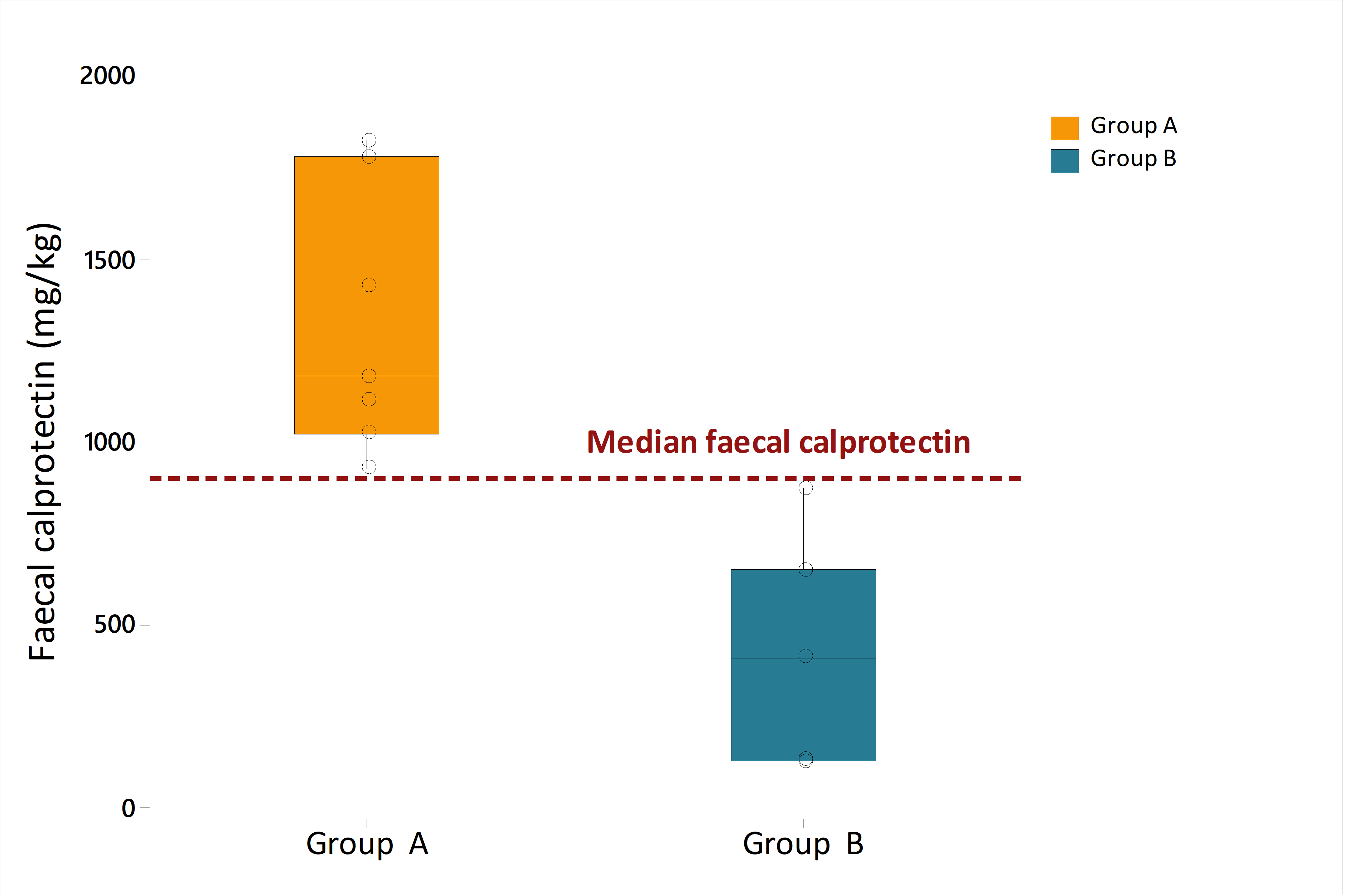

Supplement: Supplementary file 3 — Additional file 3: Fig. S3. (.tif) Stratification of patients based on the median faecal calprotectin (900 mg/kg) of the entire group at food reintroduction. Group A (n=7): above median faecal calprotectin, Group B (n=7): below median faecal calprotectin. [file 12876_2021_2029_MOESM3_ESM.tif]
