# Supplementary material for: Dietary triggers of gut inflammation following exclusive enteral nutrition in children with Crohn’s disease: a pilot study
Source: BMC Gastroenterol. 2021 Dec 3;21:454. doi: 10.1186/s12876-021-02029-4 (PMC8642954; doi:10.1186/s12876-021-02029-4)
Supplement: Supplementary file 4 — Additional file 4. Supplementary Tables 1, 2, 3. [file 12876_2021_2029_MOESM4_ESM.docx]

Supplementary Table 1. Demographic, anthropometric, and clinical disease characteristics in the two groups.

|  | Group A (n=7) | Group B (n=7) | p value |
| --- | --- | --- | --- |
| Gender (female, n [%]) | 2 (28.6) | 2 (28.6) | 1.0 |
| Age at diagnosis, y | 10.3 (8.7, 14.6) | 12.1 (11.6, 14.9) | 0.404 |
| Age at diagnosis, n (%) |  |  | 0.28 |
| A1a | 4 (57.1) | 2 (29.5) |  |
| A1b | 3 (42.9) | 5 (71.4) |  |
| Weight (kg) | 39.9 (27.4, 47.1) | 41.7 (31.6, 53.1) | 0.374 |
| Weight (z score) | -0.34 (-0.83, 0.06) | -0.09 (-0.41, -0.03) | 0.791 |
| Height (z score) | -0.34 (-0.83, 0.09) | -0.22 (-1.41, 0.78) | 0.941 |
| BMI (z score) | -0.20 (-0.55, 0.05) | -0.43 (-0.61, 0.46) | 0.553 |
| wPCDAI at end of EEN | 0 (0, 0) | 5 (0, 10) | 0.115 |
| First EEN course, n (%) | 6 (85.7) | 6 (85.7) | 1.0 |
| *MEN (%kcal) | 17.9 (0, 26.8) | 16.7 (0, 26.8) | 0.726 |
| MEN (%EAR) | 16.2 (0, 24.6) | 16.7 (0, 18.8) | 0.972 |
| Immunomodulators (azathioprine/methotrexate), n (%) | 5 (71.4) | 6 (85.7) | 0.515 |
| Disease location (Paris classification), n (%) |  |  |  |
| L2, L4a, B1 | 2 (28.6) | 1 (14.3) |  |
| L2, L4a, L4b, B1, p | 0 (0) | 1 (14.3) |  |
| L3, B1 | 0 (0) | 1 (14.3) |  |
| L3, B1, p | 1 (14.3) | 0 (0) |  |
| L3, L4a, B1 | 2 (28.6) | 1 (14.3) |  |
| L3, L4a, L4b, B1 | 2 (28.6) | 3 (42.9) |  |
| Days of food reintroduction prior to sample collection | 20 (14, 30) | 51 (16, 67) | 0.227 |

Group A: patients with faecal calprotectin levels above the median levels at food reintroduction, Group B: patients with faecal calprotectin values below the median levels at food reintroduction. Values are presented as medians (Q1, Q3) for continuous variables and as counts (frequencies) for categorical variables, wPCDAI: weighted Paediatric Crohn’s Disease Activity Index, MEN: Maintenance enteral nutrition; * 8 patients consumed Modulen® IBD, 1 patient Fresubin® Energy and 1 patient Ensure® plus

Supplementary Table 2. Correlations between the intake of nutrients and levels of FC at food reintroduction in all 14 patients.

| Nutrients | Coefficient | p value | Nutrients | Coefficient | p value |
| --- | --- | --- | --- | --- | --- |
| Total Energy (kcal) | 0.34 | 0.242 | Niacin (% RNI) | 0.61 | 0.02 |
| Total energy (% EAR) | 0.49 | 0.075 | Folic acid (ug) | 0.34 | 0.235 |
| Fat (g) | 0.04 | 0.927 | Folic acid (% RNI) | 0.43 | 0.124 |
| Fat (% kcal) | -0.40 | 0.159 | Vitamin B12 (ug) | 0.04 | 0.891 |
| SFA (g) | -0.14 | 0.642 | Vitamin B12 (% RNI) | 0.14 | 0.634 |
| SFA (% kcal) | -0.35 | 0.216 | Pantothenic acid (mg) | 0.37 | 0.194 |
| MUFA (g) | 0.24 | 0.4 | Biotin (ug) | -0.07 | 0.805 |
| MUFA (% kcal) | -0.03 | 0.917 | Vitamin E (mg) | -0.14 | 0.623 |
| PUFA (g) | 0.34 | 0.232 | Sodium (mg) | 0.39 | 0.166 |
| PUFA (% kcal) | 0.13 | 0.656 | Sodium (% RNI) | 0.39 | 0.166 |
| Carbohydrates (g) | 0.29 | 0.309 | Chloride (mg) | 0.38 | 0.181 |
| Carbohydrates (% kcal) | 0.03 | 0.924 | Chloride (% RNI) | 0.43 | 0.124 |
| Sugars (g) * | 0.15 | 0.615 | Potassium (mg) | 0.34 | 0.237 |
| Sugars (% kcal) | -0.19 | 0.509 | Potassium (% RNI) * | 0.20 | 0.485 |
| Dietary fibre (g) * | 0.42 | 0.132 | Calcium (mg) | 0.18 | 0.546 |
| Dietary fibre (% RNI) | 0.48 | 0.082 | Calcium (% RNI) | 0.15 | 0.605 |
| Starch (g) | 0.24 | 0.4 | Phosphorus (mg) | 0.26 | 0.368 |
| Protein (g) | 0.51 | 0.061 | Phosphorus (% RNI) | 0.41 | 0.15 |
| Protein (% kcal) | 0.50 | 0.069 | Magnesium (mg) | 0.19 | 0.517 |
| Protein (% RNI) | 0.54 | 0.047 | Magnesium (% RNI) | 0.31 | 0.276 |
| Vitamin A (ug) | -0.24 | 0.404 | Iron (mg) | 0.35 | 0.218 |
| Vitamin A (% RNI) | -0.17 | 0.57 | Iron (% RNI) | 0.35 | 0.214 |
| Vitamin D (ug)* | 0.34 | 0.24 | Zinc (mg) | 0.29 | 0.316 |
| Vitamin C (mg) | 0.26 | 0.375 | Zinc (% RNI) | 0.28 | 0.328 |
| Vitamin C (%RNI) | 0.30 | 0.295 | Copper (mg) | 0.05 | 0.862 |
| Thiamine (mg) | 0.57 | 0.033 | Copper (% RNI) | 0.21 | 0.482 |
| Thiamine (% RNI) | 0.67 | 0.006 | Iodine (ug) | -0.26 | 0.372 |
| Vitamin B2 (mg) | -0.07 | 0.808 | Iodine (% RNI) | -0.16 | 0.579 |
| Vitamin B2 (% RNI) | 0.26 | 0.368 | Selenium (ug) | 0.30 | 0.297 |
| Vitamin B6 (mg) | 0.18 | 0.539 | Selenium (% RNI) | 0.37 | 0.197 |
| Vitamin B6 (% RNI) | 0.34 | 0.24 | Cholesterol (mg) | 0.19 | 0.517 |
| Niacin (mg) | 0.52 | 0.058 |  |  |  |

Pearson correlations were performed except for variables marked with *, for which Spearman rank correlations were performed. FC: Faecal calprotectin

Supplementary Table 3. Correlations between the intake of foods and levels of FC at food reintroduction in all 14 patients.

| Variables | Coefficient | p value |
| --- | --- | --- |
| Cereals and cereal products | 0.36 | 0.213 |
| Gluten containing cereal products | 0.459 | 0.099 |
| Non-gluten containing cereal products * | -0.503 | 0.067 |
| Milk and milk products | -0.14 | 0.628 |
| Eggs and egg dishes * | -0.20 | 0.483 |
| Fat spreads * | 0.12 | 0.686 |
| Red meat * | 0.47 | 0.09 |
| Processed meat | 0.52 | 0.056 |
| Red and processed meat * | 0.62 | 0.019 |
| Meat and meat products * | 0.57 | 0.034 |
| Fish and fish products * | 0.24 | 0.407 |
| Vegetables and potatoes | 0.30 | 0.290 |
| Savoury snacks | -0.45 | 0.110 |
| Fruit * | -0.32 | 0.271 |
| Sugars, preserves and confectionery | -0.31 | 0.284 |
| Non-alcoholic beverages | 0.73 | 0.003 |
| Miscellaneous * | 0.49 | 0.077 |

Pearson correlations were performed except for variables marked with *, for which Spearman rank correlations were performed. FC: Faecal calprotectin
